# When nerves fail: A cause of respiratory failure in a diabetic patient

**DOI:** 10.22088/cjim.13.2.436

**Published:** 2022

**Authors:** Muhammad Talha Ayub, Tooba Ayub, Wajeeha Rasool, Muhammad Shoaib Khan, Muhammad Ishaq, Benjamin Mba

**Affiliations:** 1 Internal Medicine, John H. Stroger, Jr. Hospital of Cook County, IL (complete form), Chicago, USA; 2 Internal Medicine, AMITA Health Saint Francis Hospital, Evanston, IL (complete form), Evanston, USA; 3 Internal Medicine, Marshfield Clinic Health System, Marshfield, Wisconsin, USA

**Keywords:** Diabetic neuropathy, bilateral diabetic phrenic neuropathy, Diaphragmatic paresis.

## Abstract

**Background:**

Respiratory failure secondary to bilateral diabetic phrenic neuropathy is an uncommon clinical scenario. It is challenging to treat and often results in the need for long-term respiratory support.

**Case Presentation:**

We report a patient with long standing diabetes mellitus (DM) who presented with respiratory failure requiring mechanical ventilation. He was subsequently found to have reduced phrenic nerve and diaphragm compound action potential amplitude bilaterally on nerve conduction studies.

**Conclusion:**

Diabetic patients with unexplained shortness of breath should raise suspicion for diaphragmatic paresis from phrenic neuropathy.

Diabetic sensorimotor neuropathy is a commonly encountered clinical scenario. (1) Even though diabetic mononeuropathy can be common and most of the time is resolved spontaneously, bilateral phrenic nerve neuropathy is relatively uncommon and hard to cure, and most of the time it leads to the need of a long-term respiratory support. Respiratory failure secondary to bilateral diabetic phrenic neuropathy (BLDPN) is a rare, albeit important clinical presentation. (2, 3) It is difficult to diagnose and challenging to treat, especially in the presence of other comorbidities. We report a patient with long standing diabetes mellitus (DM) who presented with respiratory failure and who was subsequently diagnosed with BLDPN causing diaphragmatic paresis.

## Case presentation

A 65-year-old man with past medical history of type 2 DM for 22 years, diabetic sensory neuropathy involving feet, obesity with a BMI of 32.7 kg/m2, hypertension and heart failure with reduced ejection fraction presented with progressively worsening exercise tolerance and shortness of breath for two weeks. On admission, he was in mild respiratory distress with a respiratory rate of 28 per minute and blood oxygen saturation of 94% on room air. The patient was afebrile. Examination revealed excessive use of accessory muscles of respiration, decreased bilateral breath sounds with rales and wheezes. Chest x-ray was unremarkable for acute pulmonary edema but did show bibasilar changes suspicious for atelectasis. Laboratory investigations were significant for brain natriuretic peptide of 322 picogram per milliliter (pg/mL) (5-100 pg/ml) and hemoglobin A1c (HbA1c) of 8.2 % (4.0-6.0%). The patient was started on diuretic therapy for acute decompensated heart failure with preserved ejection fraction. Echocardiogram was unchanged when compared to prior study and left ventricular ejection fraction (LVEF) was 57%.

**Fig.1:**
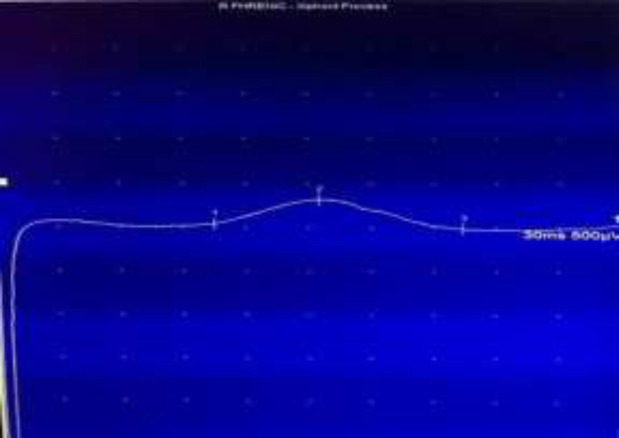
Right phrenic nerve EMG tracing with increased latency (1), decreased amplitude (2) and prolonged conduction time (3)

**Fig.2:**
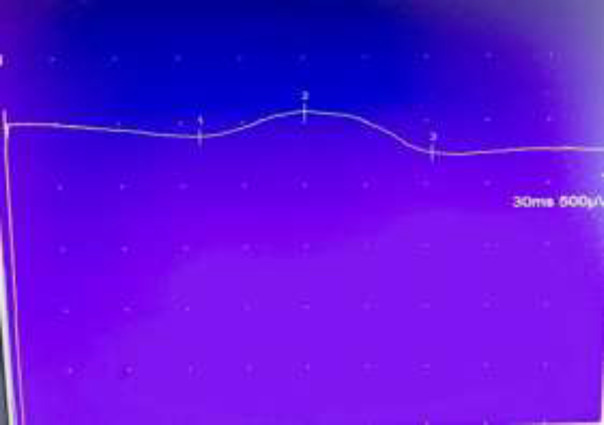
Left phrenic nerve EMG tracing with increased latency (1), decreased amplitude (2) and prolonged conduction time (3)

However, one day later, patient became disoriented and confused with his physical examination and blood gas analysis consistent with hypercapnic respiratory failure with partial pressure of carbon dioxide in arterial blood (PaCO2) of 100 millimeters of mercury (mmHg). Patient was intubated and started on mechanical ventilation. His flow volume loops were suggestive of restrictive lung disease. Even after three days of mechanical ventilation and optimal heart failure therapy, he failed a spontaneous breathing trial and remained obtunded from persistent carbon dioxide narcosis (PaCO2 of 64 mmHg). Diaphragmatic muscle palsy was suspected based on the nocturnal need for assisted ventilation and radiographic evidence of elevated diaphragmatic domes on inspiration. Nerve conduction studies showed reduced phrenic nerve and diaphragm compound action potential amplitude bilaterally (figs.1 and 2), consistent with an advanced diabetic polyneuropathy.

Patient was managed with non-invasive bi-level positive airway pressure (BiPAP) ventilation and over the course of a few days, his condition improved. He was discharged 2 weeks later on home BiPAP and was found to be maintaining his baseline level of functionality on post hospital follow up visits. He was then lost to follow-up and later died of cardiopulmonary arrest as per the spouse.

## Discussion

Diabetic neuropathy commonly manifests as distinct clinical syndromes like distal symmetric polyneuropathy, autonomic neuropathy, polyradiculopathies, focal mononeuropathies and mononeuritis multiplex.(1) Bilateral phrenic nerve neuropathy, causing respiratory failure secondary to diabetes mellitus, has been rarely reported and scarcely studied.(2, 3, 4, 5) Although the duration of diabetes is the predominant factor in determining the onset and progression of neuropathy but it has been reported in patients with pre-diabetes and mildly uncontrolled disease.

Bilateral diaphragmatic palsy presents as progressive shortness of breath which worsens in the supine position. (6, 7) Physical examination may reveal classical paradoxical abdominal wall retraction during inspiration. Diagnosis is established in the clinical context of hypercapnic respiratory failure, not otherwise explained, with suggestive findings on imaging, pulmonary function testing (PFT) and nerve conduction studies (8). Blood gas analysis may show hypercarbia with or without hypoxemia. A frontal upright chest radiograph showing characteristic smooth elevation of the hemi-diaphragm is a sensitive marker (90%) for diaphragmatic muscle weakness but it is nonspecific (44%) (9, 10). PFT will typically show low forced vital capacity with significant reduction in supination, decreased minimal inspiratory pressure and normal maximal expiratory pressure (11). Measurement of trans-diaphragmatic pressures is the gold standard for diagnosis of diaphragmatic palsy. Diaphragmatic electromyography (EMG) with nerve conduction studies is a more invasive test which shows reduction in the compound muscle action potential and prolongation of nerve conduction time. (12) 

Nocturnal BiPAP has been well studied in patients with bilateral diaphragmatic weakness secondary to neurodegenerative diseases (13, 14) and is the supportive treatment, especially in patients with an arterial PaCO2 of 48 mmHg or higher or maximal inspiratory pressure less than -60 centimeters of water. Surgical phrenic nerve transfer and pacing requires further studies. (15) Prognosis is guarded considering progressive and irreversible nature of neuropathy. 

In conclusion diaphragmatic paresis from phrenic nerve neuropathy is a rarely reported complication of long-standing diabetes mellitus. Diabetic patients with unexplained shortness of breath should raise suspicion for diaphragmatic paresis from phrenic neuropathy. 
